# VO Cluster-Stabilized H_2_O Adsorption on a TiO_2_ (110) Surface at Room Temperature

**DOI:** 10.1021/acs.jpcc.2c06202

**Published:** 2022-10-18

**Authors:** Xiao Tong, Scott P. Price, Jeremy C. Robins, Claron Ridge, Hyun You Kim, Paul Kemper, Horia Metiu, Michael T. Bowers, Steven K. Buratto

**Affiliations:** Department of Chemistry and Biochemistry, University of California, Santa Barbara, Santa Barbara, California 93106-9510, United States

## Abstract

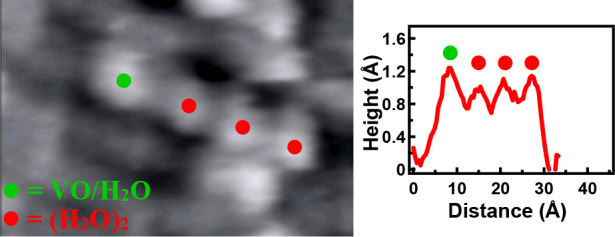

We probe the adsorption of molecular H_2_O on a TiO_2_ (110)-(1 × 1) surface decorated with isolated VO clusters using ultrahigh-vacuum scanning tunneling microscopy (UHV-STM) and temperature-programmed desorption (TPD). Our STM images show that preadsorbed VO clusters on the TiO_2_ (110)-(1 × 1) surface induce the adsorption of H_2_O molecules at room temperature (RT). The adsorbed H_2_O molecules form strings of beads of H_2_O dimers bound to the 5-fold coordinated Ti atom (5c-Ti) rows and are anchored by VO. This RT adsorption is completely reversible and is unique to the VO-decorated TiO_2_ surface. TPD spectra reveal two new desorption states for VO stabilized H_2_O at 395 and 445 K, which is in sharp contrast to the desorption of water due to recombination of hydroxyl groups at 490 K from clean TiO_2_(110)-(1 × 1) surfaces. Density functional theory (DFT) calculations show that the binding energy of molecular H_2_O to the VO clusters on the TiO_2_ (110)-(1 × 1) surface is higher than binding to the bare surface by 0.42 eV, and the resulting H_2_O–VO–TiO_2_ (110) complex provides the anchor point for adsorption of the string of beads of H_2_O dimers.

## Introduction

Metal and metal oxide nanoparticles supported on TiO_2_ are highly active catalysts for the selective oxidation of organic compounds.^1−16^ For example, gold nanoparticles supported on titania catalyze the oxidation of propene to propylene oxide,^3−6^ whereas vanadia nanoparticles supported on the TiO_2_(110) surface catalyze the oxidative dehydrogenation of methanol to formaldehyde.^7−16^ To understand the catalytic chemistry at the molecular level, it is important to understand the interaction of the supported catalysts with the various molecular species participating in the reaction. These interactions are generally probed individually under ultrahigh-vacuum (UHV) conditions using model catalysts supported on clean single-crystal surfaces. One of the most important molecules is water; it is omnipresent and often actively participates in the chemistry, as is the case for the oxidative dehydrogenation of methanol where water is a product of the reaction.

The interaction of water with the reduced TiO_2_(110) surface has been studied extensively at a wide range of temperatures.^17−33^ Water reacts with the reduced TiO_2_(110) surface at bridging oxygen vacancies over a wide temperature range from 150 K to RT, resulting in dissociative adsorption.^17−27^ The dissociation reaction produces an OH group that fills the vacancy and a hydrogen adatom that binds to an adjacent bridging oxygen, forming the so-called double hydroxyl. This double hydroxyl is readily visible in scanning tunneling microscopy (STM) as a bright spot centered along the bridging oxygen row and slightly elongated in the [001] direction.^20−23,25^ These OH groups can be recombined to form water by heating the surface, producing a characteristic peak in the water TPD spectrum at 500 K.^26,27^

Molecular water is not observed on the reduced TiO_2_(110) surface at RT using STM.^22,23^ This is due to the short residence time of molecular water at temperatures higher than 250 K.^23^ It is, however, possible to observe molecular water on the reduced TiO_2_(110) surface at low temperature.^23−25,33^ Water TPD has shown two desorption states for molecular water at approximately 195 and 295 K,^26,27^ which are attributed to desorption of the second layer and the first layer water, respectively. It is important to note that molecular water desorbs at temperatures below 300 K, a further indication that molecular water is not observed on the reduced surface above 300 K. Redhead analysis of the first layer desorption peak (*T* = 295 K) of the water TPD spectrum suggests that the adsorption energy of this layer is ∼0.8 eV.^26^ STM images of a submonolayer coverage of water adsorbed on the reduced surface at 150 K show that the molecular water forms string of beads features centered along the 5c-Ti rows.^23,33^ Detailed analyses of the STM images coupled with DFT calculation have suggested that string of beads features are composed primarily of water dimers for moderate water coverages.^24,25^ The water dimer was shown to be stable on the surface with a binding energy of 1.86 eV (0.93 eV/molecule),^24,25^ which is slightly higher than the value predicted by the TPD data. The water dimer exhibits a characteristic STM signature: a single bright spot centered along the 5c-Ti row, 1.0 Å in height, and a full width at half-maximum (FWHM) equal to 6 Å (twice the distance between adjacent Ti atoms along the 5c-Ti row).^24^ Finally, the water dimer was shown to have much higher mobility than an individual water molecule, which implies that at sufficiently low water coverage the water dimer is the most abundant water species on the surface. As the water dimers diffuse along the 5c-Ti rows, they coalesce into weakly interacting strings oriented along the 5c-Ti rows.^24^ The string of beads features are observed on the reduced surface for temperatures below 250 K.^23,33^ At temperatures above 250 K the only evidence of water adsorption observed in STM is the water that dissociates at the oxygen vacancies to form OH groups.^23^

Recently the interactions between water and other coadsorbates on TiO_2_(110) surfaces have been reported. H_2_O readily reacts with oxygen adatoms (denoted O_a_) on TiO_2_(110).^32,34,35^ Molecular water dissociates in the presence of O_a_, resulting in the binding of two hydroxyl groups along the 5c-Ti row.^32^ The recombination and desorption of these hydroxyls is observed in TPD as a high temperature tail of the monolayer desorption peak at around 350 K.^26^ This tail in the desorption peak is similar in temperature and shape to that observed for TiO_2_(110) surfaces that were irradiated with low-energy electrons after exposure to water at low temperature. This tail has been attributed to recombinative desorption of hydroxyls formed on the 5c-Ti rows as a result of the dissociation of water in the first layer during the electron bombardment process.^36^ The interaction of water with gold-covered TiO_2_(110) has also been studied with STM,^37,38^ TPD, XPS, and ion scattering.^39,40^ In these experiments it was shown that water will react with Au adatoms bound to the bridging oxygen vacancies by first dissociating, then displacing the Au atoms from the vacancies, and last forming OH–Au–OH complexes.^37^ No molecular water was observed in STM images,^37^ and no additional TPD states were observed for water adsorbed to Au-covered TiO_2_(110).^39,40^

In the work described here we study the interaction of water with a rutile TiO_2_(110)-(1 × 1) surface containing a submonolayer coverage of mass-selected vanadium oxide (VO) clusters at 300 K under UHV conditions, using a combination of STM and TPD. We compare these results to water interacting with the bare TiO_2_(110) surface at 300 K as well as to TiO_2_(110) surfaces decorated with clusters of V, V_2_, and VO_2_ stoichiometry. We investigate the stability of VO bound 1-D water chains and their influence on water dimer dynamics at RT using both time-lapsed STM and density functional theory (DFT) calculations. Details of these investigations on the interaction of H_2_O with decorated TiO_2_(110) surfaces are discussed.

## Experimental Methods

The clean TiO_2_(110)-(1 × 1) surface was prepared by multiple cycles of Ar^+^ ion sputtering (1 keV, 20 min) and annealing (∼850 °C, 10 s) and then examined by STM. The typical vacancy concentration observed by STM for surfaces used in this study is 10%. VO deposition is accomplished using a home-built mass-selected cluster source described in detail elsewhere.^45^ Vanadium oxide clusters are created by laser ablation of a vanadium metal target in an argon expansion gas seeded with 20% O_2_.^43^ Positive ions are extracted, accelerated, and focused using several steering lenses before being size-selected by a magnetic-field analyzer. The size-selected VO^+^ ion beam has a flux of ∼1 nA/cm^2^. This beam is directed into a UHV chamber containing the TiO_2_(110)-(1 × 1) substrate which is held at +190 V to decelerate the VO^+^ ions. Biasing the titania sample allows VO^+^ ions to be soft-landed onto the surface with incident kinetic energy of <2.0 eV per atom. The base pressure of the deposition chamber is <4 × 10^–10^ Torr during deposition. Exposure times of 90 min at RT result in a surface coverage of ∼0.02 ML as determined from the STM images. Monolayer coverage is defined as one VO cluster or one water molecule per TiO_2_ unit cell (5.2 × 10^14^ cm^–2^).

After deposition, the VO-decorated surface is transferred to the STM chamber and imaged using a RHK UHV350 SPM at a base pressure of 1 × 10^–10^ Torr. Empty state STM images of the surface are acquired at 300 K in constant-current mode with a sample bias of +1.5 V and a tunneling current of 0.1–0.2 nA. For H_2_O deposition, deionized Millipore H_2_O was purified by several freeze–pump–thaw cycles and introduced into the UHV STM chamber through a calibrated leak valve. Water TPD is performed by moving the sample into position about 1 mm in front of a 3 mm wide skimmer cone aperture of a mass spectrometer (RGA200, Stanford Research Systems) and heating from RT to 600 K at a rate of 1 K/s.

Details of our density functional theory (DFT) calculations for TiO_2_(110)-supported vanadia clusters can be found in our previous work.^13,14^ Briefly, DFT+*U* (*U* = 3.4 eV) calculations were performed on a TiO_2_(110) slab with a thickness of 12 atomic layers and a [4 × 1] surface supercell using plane-wave code VASP with the Perdew–Wang functional.

## Results and Discussion

**Figure 1:**
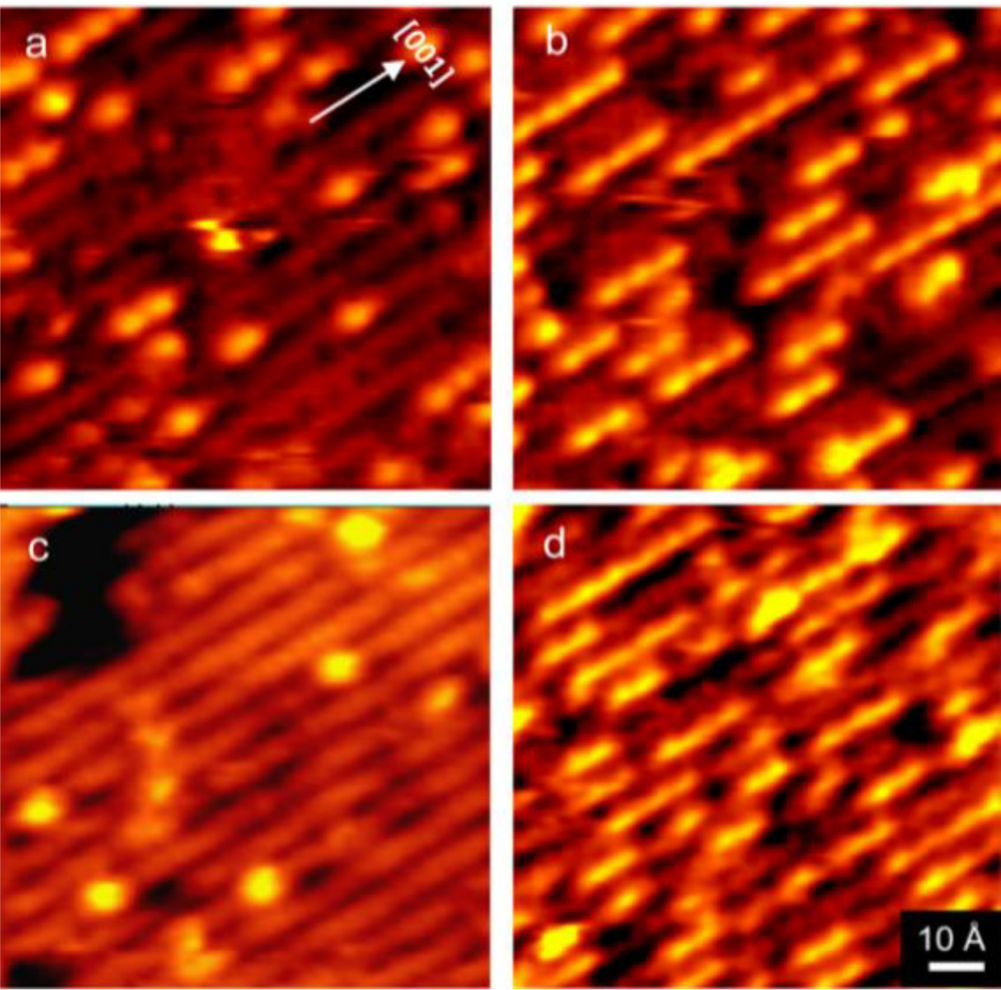
Room-temperature STM images of the VO-decorated TiO_2_ surface: (a) 12 h after deposition, (b) after exposing the surface from (a) to 2.5 langmuirs of water, (c) 2 h after annealing the surface from (b) to 600 K for 30 s, and (d) after exposing the surface from (c) to 2.5 langmuirs of water.

**Figure 2:**
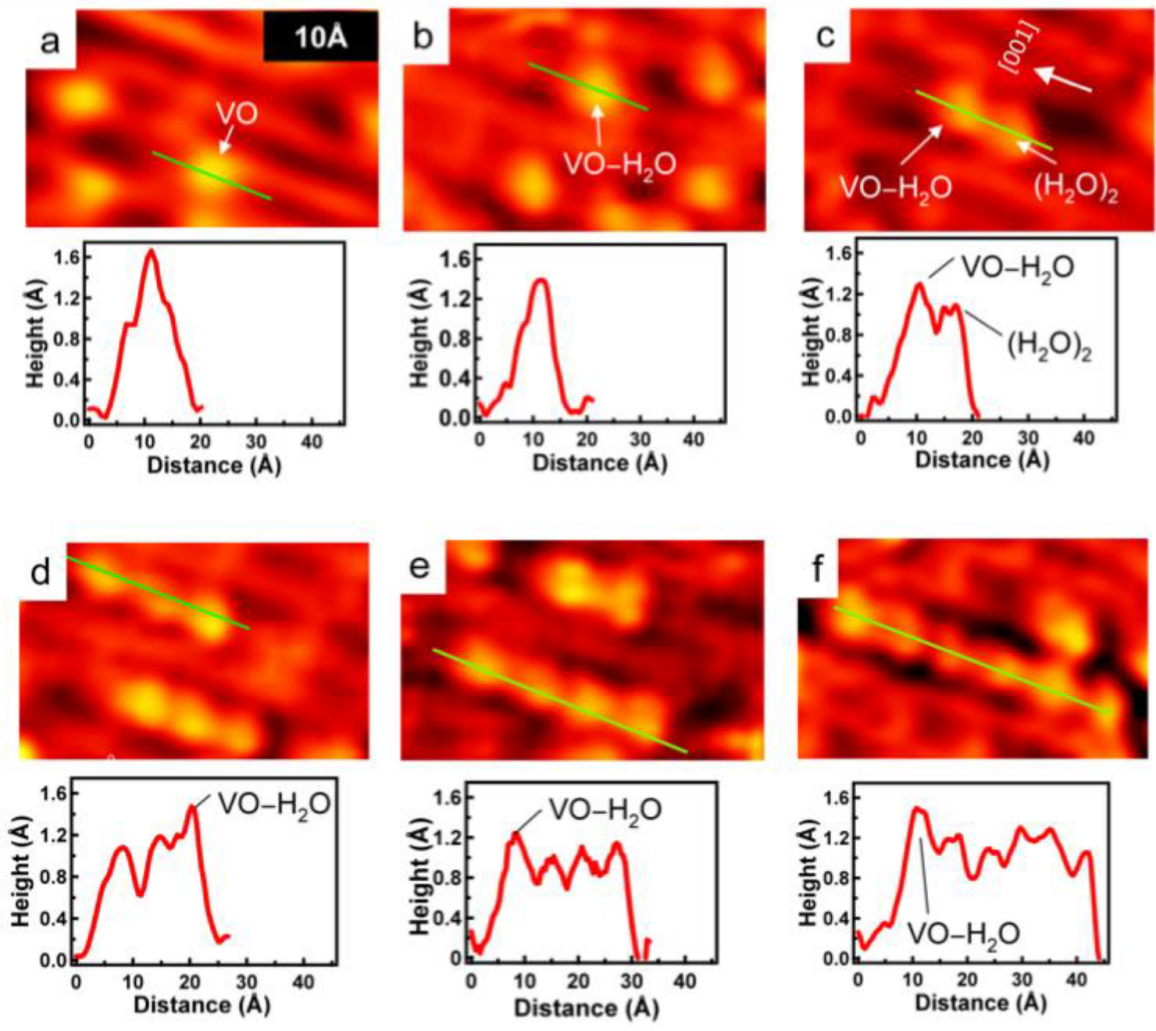
High-resolution STM images exhibiting water strings of beads of varying length. (a) Isolated VO cluster. (b) Isolated VO–H_2_O complex. Strings with two (c), three (d), four (e), and six (f) beads are also shown. Line scans taken along the [001] direction across the features are presented with each image. For images showing more than one string with a given length, a green line is shown to indicate which string is examined in the line scan.

The interaction of H_2_O with the VO-decorated TiO_2_(110)-(1 × 1) surface is illustrated in the STM images of Figure 1. The STM image of Figure 1a shows the VO-decorated TiO_2_(110)-(1 × 1) surface prior to the introduction of H_2_O vapor into the UHV chamber. The image presented in Figure 1a was acquired 12 h after the deposition of the VO clusters on the TiO_2_ surface. It was the first image taken of the VO-decorated surface. The 12 h delay is due to the time required for our UHV chamber to return to the base pressure (2 × 10^–10^ Torr) and to transfer the sample into the STM chamber. In this image isolated bright spots appear superimposed over alternating bright and dark stripes along the [001] direction, the characteristic STM features of rutile TiO_2_(110)-(1 × 1).^41,42^ The bridging oxygen (O_br_) atom rows appear dark in the empty state STM images while the 5-fold coordinated titanium (5c-Ti) atom rows appear bright. The isolated bright spots are attributed to the VO clusters.^43^ As we have discussed in our previous work, the VO cluster binds to the TiO_2_(110)-(1 × 1) surface with the V atom in an upper 3-fold hollow site and the O atom bound to an adjacent 5c-Ti atom.^43^ The STM image of the VO cluster shows a bright spot asymmetric to the O_br_ and 5c-Ti rows, slightly elongated in the [001] direction with a height of 1.6 Å. A high-resolution image of VO clusters is shown in Figure 2a. Exposing the VO-decorated TiO_2_(110)-(1 × 1) surface to 2.5 langmuirs of H_2_O at RT results in significant adsorption of water as shown in the STM image of Figure 1b. The adsorbed H_2_O appears as string of beads features oriented parallel to the [001] direction and positioned directly above the 5c-Ti rows. These strings of beads are strikingly similar to the features previously observed by the Thornton group for H_2_O adsorbed to bare TiO_2_(110)-(1 × 1) at 150 K.^23^ The adsorbed H_2_O is readily removed by annealing the surface at 600 K for 30 s. An STM image of the surface that results from this annealing is shown in Figure 1c. All of the string–bead features are removed, leaving a surface covered with isolated bright spots similar to those observed in the image of Figure 1a. The feature density in the image of Figure 1c is ∼0.02 ML, which is the coverage expected from the original deposition of VO. This implies that the VO clusters are stable up to the annealing temperature of 600 K. We see no evidence of sintering of the VO clusters. In fact, we are unable to remove the VO clusters from the surface simply by heating. To remove these clusters completely requires several cycles of Ar^+^ sputtering followed by annealing at 850 °C. It is interesting to note that the feature density of Figure 1a is much higher than that of Figure 1c. In addition, several features in the image of Figure 1a are reminiscent of the shorter string of beads features of Figure 1b. It is likely that the image of Figure 1a contains some water that was adsorbed from the background during the delay between the deposition of the VO clusters and the initial STM image, which was 12 h. These additional features are the small strings of beads (containing two or three bead features) that appear in the image of Figure 1a. The VO-decorated surface is an efficient getter for background water—we have observed the formation of string–beads on VO-decorated TiO_2_(110)-(1 × 1) surfaces that have only been exposed to the base pressure (2 × 10^–10^ Torr) for several days (data not shown).

Exposing the surface of Figure 1c to an additional 2.5 langmuirs of H_2_O results in adsorption of water and regeneration of the strings of beads as seen in the image of Figure 1d. It is possible to cycle between the surfaces of Figures 1c and 1d by alternating between adsorption of H_2_O by exposing the surface to 2.5 langmuirs of H_2_O to form the string–beads and removal of the H_2_O by annealing at 600 K to reproduce the VO-decorated surface. We have repeated this cycle several times, indicating that the process of water adsorption followed by annealing is completely reversible.

To better understand the formation of the string of beads features shown in Figure 1, we have studied the initial stages of the water adsorption to the VO-decorated TiO_2_(110)-(1 × 1) surface. We have observed that the VO clusters serve as the anchor point for the adsorption of H_2_O and the growth of the strings of beads. This is illustrated by the data of Figure 2, which shows high-resolution STM images of string–beads of various lengths. Line cuts of the strings of beads are also presented for each image to illustrate both the height of the beads as well as the position of the beads relative to the 5c-Ti atoms. An isolated VO cluster prior to water adsorption is shown in the STM image of Figure 2a. The VO cluster is positioned asymmetrically with respect to the O_br_ and 5c-Ti rows as discussed previously^43^ and has a height of 1.6 Å as seen from the line cut. The orientation and height of STM feature of Figure 2a are a defining signature of a bound VO cluster. Exposing the VO-decorated surface to a small amount of water (2.5 langmuirs) results in the isolated spots shown in the STM image of Figure 2b. These isolated spots differ from those observed in Figure 2a in two ways. The spots observed in Figure 2b are positioned nearly symmetrically with respect to the 5c-Ti rows and each have a height of 1.4 Å, a subtle but significant difference from the VO clusters observed in Figure 2a. We can measure height in our STM to ±0.1 Å, which is less than the height difference of two spots in Figure 2a,b. We assign the isolated structure of Figure 2b as a single water molecule adsorbed to a single VO cluster. It is important to note that STM alone cannot resolve the nature of the adsorbed water—whether it is bound as molecular water or dissociated water. A detailed structural model of the resulting VO–H_2_O complex derived from a comparison of the STM images to density functional theory (DFT) calculations attributes this feature to an undissociated water molecule adsorbed to the VO cluster supported on the TiO_2_ (110)-(1 × 1) surface. These calculations will be presented and discussed later.

As the water adsorption to the VO-decorated surface progresses, the strings of beads (similar to those shown in Figure 1b,d) begin to appear and increase in length. Figure 2c shows a two-bead structure, which is symmetric with respect to the 5c-Ti rows. The relative heights and positions of the two features are shown in the line cut. The feature on the left has a height of 1.4 Å while the feature on the right is slightly shorter with a height of 1.2 Å. We attribute the taller feature to the VO–H_2_O complex as it has the same STM signature as that of Figure 2b. The shorter feature we attribute to a water dimer, the dominant form for molecular water on TiO_2_ as suggested by Matthiesen et al.^24^ In addition, the two peaks are separated by a distance of ∼6 Å. The distance between adjacent Ti atoms along the 5c-Ti row is only 3 Å, which implies that the two features of Figure 2c are separated by two Ti atoms. This is the same spacing observed previously for string–beads of water dimers on TiO_2_ at 150 K.^24^ Finally, the full width at half-maximum of the shorter bead feature of Figure 2c is 6 Å, which is the same as that observed for the water dimer by Besenbacher and co-workers.^24^

Longer string–beads are shown in STM images of Figure 2d–f. The image of Figure 2d shows a string with three features. In this image the VO–H_2_O complex is the rightmost feature, which is the tallest feature (1.4 Å) as seen in the line cut. The other two features have heights of 1.2 Å and are separated by 6 Å, the distance of two 5c-Ti atoms. The STM image of Figure 2d shows a string of four features. The VO–H_2_O complex is at the far left with the characteristic height of 1.4 Å. The remaining three features are separated by two 5c-Ti atoms (6 Å) and have heights of 1.2 Å, similar to the separation between the water dimer beads in the images of Figure 2c,d. The STM image of Figure 2f shows a string of six features with the VO–H_2_O complex at the far left. The remaining five features are again separated by two 5c-Ti atoms and have heights of 1.2 Å. The string of beads feature of Figure 2f is an example of the longest string we have observed.

As water continues to adsorb to the VO-decorated surface, we observe string of beads features that are not anchored by a VO–H_2_O complex. These strings of beads appear as branches of strings of beads that have a VO–H_2_O complex as an anchor. Additional water adsorption occurs up to a saturation coverage of 0.38 ML (2.0 × 10^14^ water molecules/cm^2^). This observation implies that while the strongest adsorption occurs for water anchored by a VO–H_2_O complex, the presence of the VO–H_2_O anchored string of beads features stabilizes additional string of beads features on adjacent 5c-Ti rows. The adsorption of water on the VO-decorated TiO_2_ surface is thus self-limiting to the saturation coverage of 0.38 ML, which is >10 times the coverage of VO. We have not been able to observe water adsorption larger than saturation coverage for a single layer.

**Figure 3:**
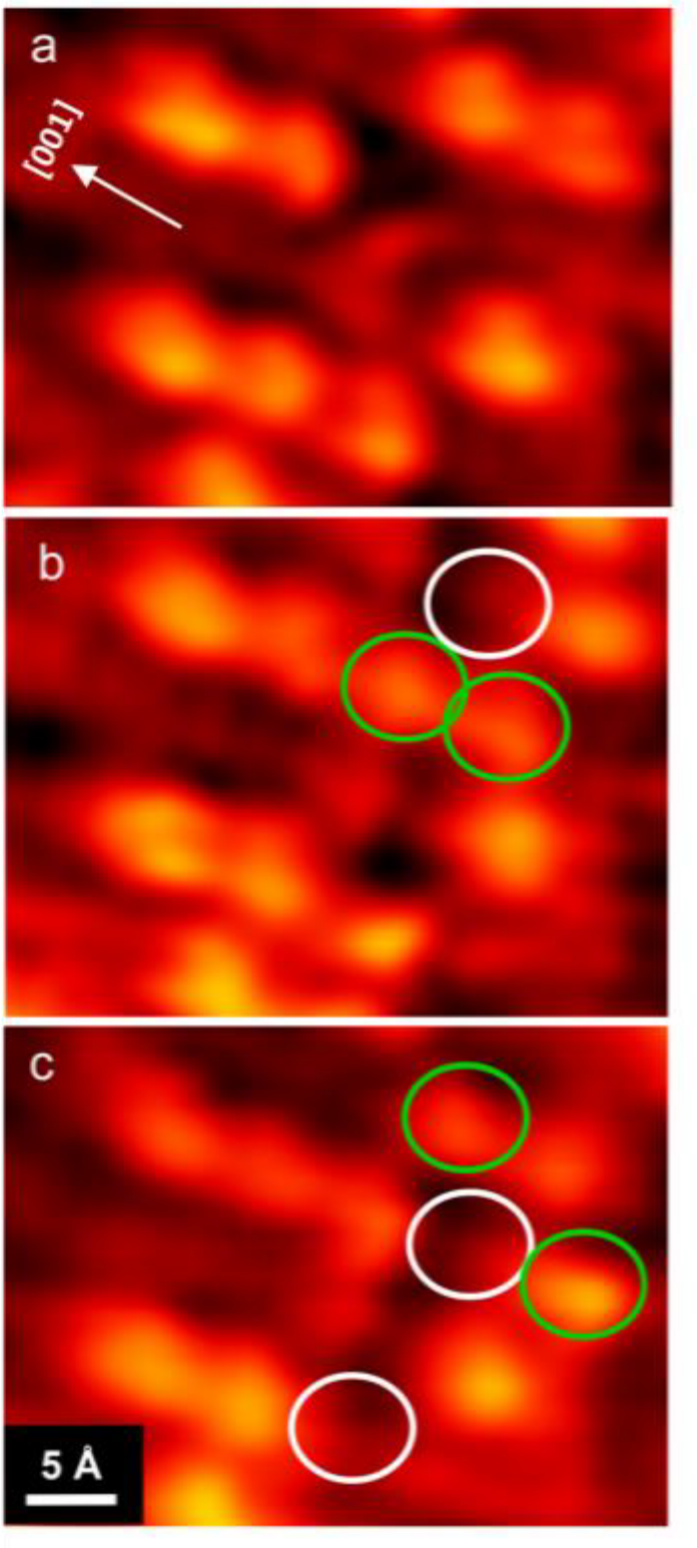
High-resolution STM images of the same area over time. (a) The surface at time *t*_0_, showing a total of nine beads. The surface at time (b) *t*_0_ + 5 min and (c) *t*_0_ + 11 min, both showing a total of 10 beads. Water dimers that disappeared between subsequent images are highlighted with white circles, while new water dimers that appeared between subsequent images are marked with green circles.

We have observed that the longer strings of beads are not stable over time as seen in the time-lapsed STM images of Figure 3. The initial STM image of Figure 3a shows several bead features with a total of nine beads. Five minutes after acquiring the image of Figure 3a, the same area of the sample now contains 10 beads, as shown in Figure 3b. Two new beads appear as indicated by the green circles, creating a string of four beads. Initially, this same string had only two beads as seen in Figure 3a. The white circle in Figure 3b indicates the loss of a bead feature originally present in the image of Figure 3a. The STM image of Figure 3c is also of the same area of the sample acquired 6 min after the image of Figure 3b (11 min after the image in Figure 3a). Again, there are a total of 10 beads in this image, but four changes have occurred between the image of Figures 3b and 3c. Two beads have appeared as indicated by the green circles, and two beads have been lost as indicated by the white circles. The appearance and disappearance of the bead features indicate that while the water is adsorbed onto the VO-decorated surface at room temperature and can be imaged by STM, the adsorption is weak, and there is significant mobility of the water on the surface.

**Figure 4:**
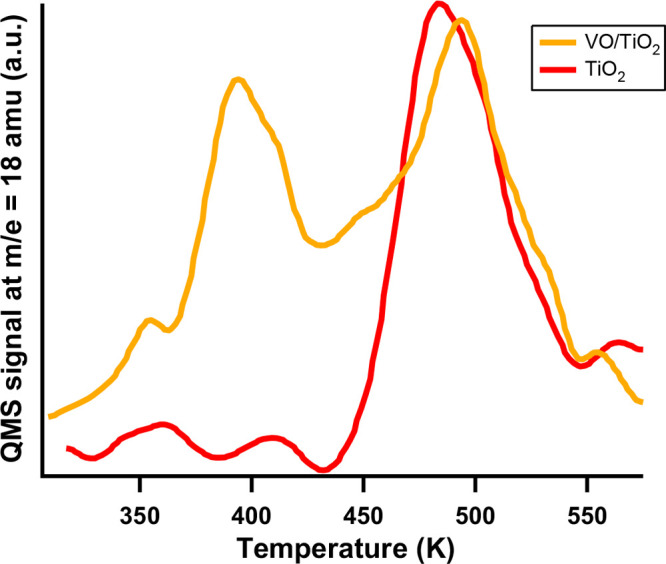
Water TPD spectra from the bare (red line) and 2% ML VO-decorated (orange line) TiO_2_(110) surface following exposure to 2.5 langmuirs of H_2_O at room temperature.

Figure 4 shows TPD spectra of water from the bare TiO_2_(110) surface (red line) and the VO-decorated surface (orange line). The TPD spectrum from the bare TiO_2_(110) surface exhibits a single peak located at 500 K, which, as discussed previously, has been assigned to recombination of two hydroxyls.^27,28^ In contrast, the TPD spectrum for the VO-decorated surface shows three desorption states: one at 395 K with a broad leading edge extending to room temperature; a second, smaller, peak at 445 K; and the recombination peak at 500 K. On the basis of the STM results discussed above, we assign the peak at 395 K to desorption of molecular water from the 5c-Ti rows that form the string of beads features. The broad leading edge of the peak is attributed to desorption of weakly bound water molecules located away from the VO anchor point. To assign the peak at 445 K, the TPD peak areas were integrated and referenced to the recombination of water from vacancies (10% vacancy concentration). The area of the peak at 395 K corresponds to a water coverage of 0.071 ML. This is consistent with desorption of water dimers since this is approximately twice the water coverage of the deposited VO clusters. For the peak at 445 K, the calculated water coverage is 0.014 ML, which is very close to the coverage of deposited VO clusters (0.02 ML). Therefore, we assign the peak at 445 K to desorption of single water molecules bound to the VO clusters. A Redhead analysis,^44^ assuming a pre-exponential factor of 10^12^ gives adsorption energies of 1.0 and 1.2 eV per water molecule for the peaks at 395 and 445 K, respectively.^26^ Combining the adsorption energies obtained from our TPD results with the calculated adsorption energy for water bound to the bare TiO_2_(110) surface of 0.8 eV^28^ allows us to conclude that the preadsorbed VO molecules increase the binding energy of water on the TiO_2_(110) surface by 0.2–0.4 eV.

**Figure 5:**
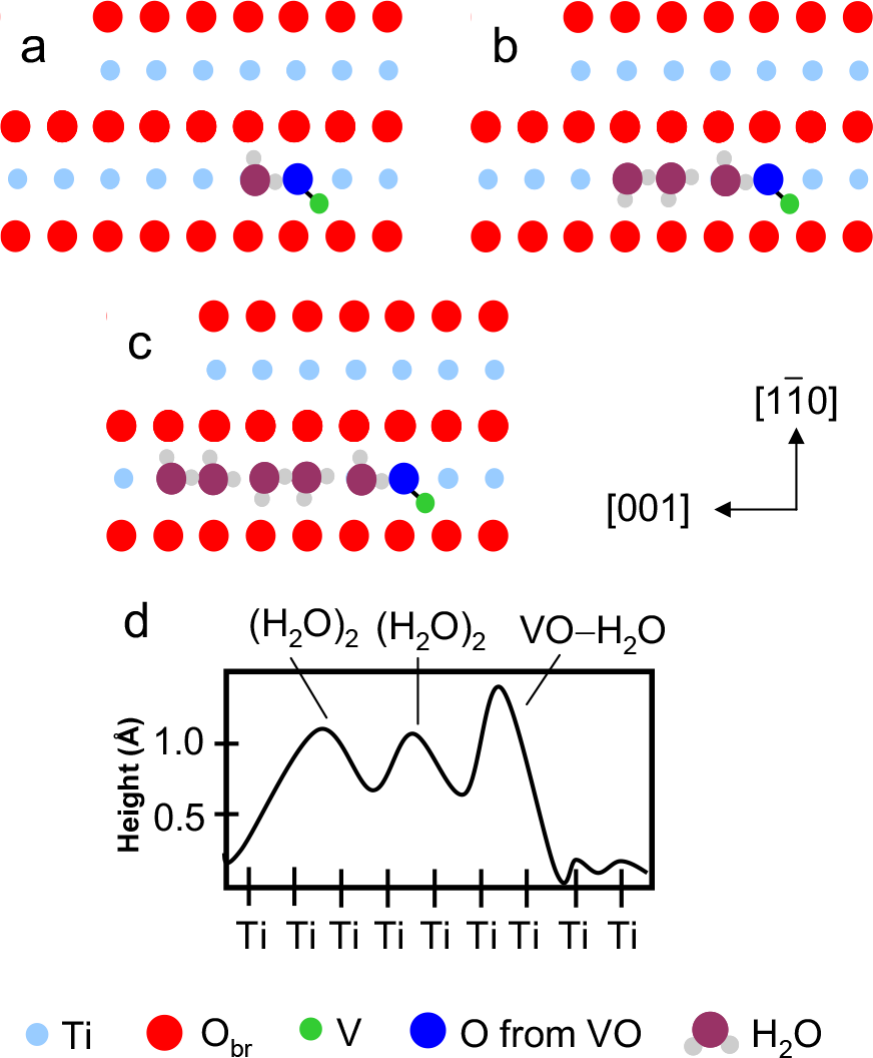
Atomistic structural models for water adsorption to the VO-decorated TiO_2_ surface. Top-view models of the titania surface (a) with a single water molecule bound to a VO cluster, (b) after adsorption of the first water dimer, and (c) after adsorption of the second water dimer. (d) A simulated line cut along the string of beads shown in (c).

We have used density functional theory (DFT) to model the interaction of H_2_O with a VO decorated TiO_2_ (110)-(1 × 1) surface as shown in Figure 5. The ball and stick model from the DFT calculations is including in Figure S2. The cartoon model of Figure 5 is based on the results of the DFT calculations and is presented so that the features can be directly compared with our STM images. The lowest energy structure for a single H_2_O molecule adsorbed to VO/TiO_2_(110) is shown in Figure 5a. The H_2_O molecule is centered on the 5c-Ti row the TiO_2_(110) surface with the oxygen atom bound to the 5c-Ti atom adjacent to the oxygen atom of the VO cluster. The hydrogen atoms are arranged such that one H atom forms a hydrogen bond to the oxygen atom of the VO and the other H atom points toward a bridging oxygen atom as shown. This H_2_O molecule has a binding energy of 1.02 eV, which is 0.22 eV higher than the binding energy of a H_2_O molecule to the bare TiO_2_(110) surface.^28^ It should be noted that the lowest energy structure for the H_2_O–VO complex shown in Figure 5a is not the only low-energy structure predicted by DFT. The pseudodissociated structure (not shown)^24,26^ in which the hydrogen atom is transferred from the H_2_O molecule to the VO has a binding energy only 0.024 eV lower than the structure shown in Figure 5a. The structure of the pseudo-dissociated structure is nearly identical with the structure of Figure 5a and differs only in the position of the H atom forming the hydrogen bond between the H_2_O and the VO. In the pseudo-dissociated structure this atom is closer to the VO than is represented in Figure 5a.

The H_2_O–VO complex depicted in Figure 5a acts as an anchor point for the subsequent adsorption of water. The structure of Figure 5b shows the adsorption of a water dimer to the H_2_O–VO complex. A large fraction of the water on the TiO_2_(110) surface has been proposed to be in the form of a water dimer. The water dimer of Figure 5b is the same structure predicted by Matthiesen et al.,^24^ has a binding energy of 1.84 eV, and is highly mobile along the 5c-Ti rows. In our adsorption model the H_2_O–VO complex acts as a barrier to diffusion and provides a binding site through hydrogen bonding to the water molecule of the H_2_O–VO complex. The binding of the water dimer is directional. The position of the O atom of the VO cluster defines the orientation of the H_2_O–VO complex as well as the direction for adsorption of water dimers. Additional water dimers adsorbed to other water dimers as depicted in the structure of Figure 5c, which shows two water dimers bound to the H_2_O–VO complex. The aggregation of water dimers into larger structures along the 5c-Ti rows has been reported previously for water adsorbed to TiO_2_(110) at 160 K,^23^ and our model for the additional aggregation of water dimers to the H_2_O–VO complex is similar.

The (H_2_O)_2_–H_2_O–VO/TiO_2_(110) structure shown in Figure 5b is represented in the STM data as two bright spots centered on the 5c-Ti row (see, for example, the image of Figure 2c). The taller bright spot is from the H_2_O–VO complex, and the second bright spot is the water dimer. The two spots are separated by 6 Å, the distance between the center of the H_2_O–VO complex and the center of the water dimer. This distance is equivalent to twice the distance between Ti atoms in the 5c-Ti rows. Similarly, the (H_2_O)_2_–(H_2_O)_2_–H_2_O–VO/TiO_2_(110) structure shown in Figure 5c is represented in the STM data as three bright spots centered on the 5c-Ti row (see, for example, the image of Figure 2d). A simulated STM line cut along the string of beads from Figure 5c is presented in Figure 5d and can be compared to the experimental STM line cut shown in Figure 2d.

## Conclusion

We have shown, for the first time, that RT adsorption of molecular water is possible on a rutile TiO_2_(110)-(1 × 1) surface containing a submonolayer coverage of mass-selected vanadium oxide (VO). The adsorption results in string–beads of water dimers bound to the 5c-Ti rows and anchored by VO molecules. This adsorption is strongly dependent on the composition of the vanadium species and is only observed for the VO-decorated surface. The RT adsorption does not occur for bare TiO_2_(110) surfaces or for surfaces decorated with V, V_2_, and VO_2_ (see the Supporting Information). We have determined that the first water (bound to the VO cluster) has a binding energy of 1.2 eV and subsequent water molecules are bound as dimers with a binding energy of 1.0 eV/H_2_O molecule. These adsorption energies are close to those predicted by DFT calculations. These results will be useful in developing a more detailed understanding of the role of H_2_O in the catalytic activity of supported vanadium clusters on oxide surfaces.

## Supporting Information Available

STM investigations on the adsorption and stabilization of water on rutile TiO_2_ surfaces, decorated with size-selected V, V_2_, and VO_2_ clusters were performed and shown in Figure S1; ball and stick models of the lowest energy DFT structures of VO, VO + 1H_2_O, VO + 2H_2_O, and VO + 3H_2_O bound to a rutile TiO_2_ surface are shown in Figure S2 (PDF)
