# Supplementary material for: VO Cluster-Stabilized H2O Adsorption on a TiO2 (110) Surface at Room Temperature
Source: J Phys Chem C Nanomater Interfaces. 2022 Oct 18;126(42):17975–82. doi: 10.1021/acs.jpcc.2c06202 (PMC9619923; doi:10.1021/acs.jpcc.2c06202)
Supplement: Supplementary file 1 — jp2c06202_si_001.pdf [file jp2c06202_si_001.pdf]

VO Cluster-Stabilized H_2O Adsorption on a TiO_2 (110) Surface at Room Temperature

Xiao Tong[a], Scott P. Price, Jeremy C. Robins, Claron Ridge[b], Hyun You Kim[c], Paul Kemper, Horia Metiu, Michael T. Bowers, and Steven K. Buratto *

Department of Chemistry and Biochemistry, University of California, Santa Barbara, CA 93106-9510, USA

[a] Current Address: Brookhaven Natl Lab, Center for Functional Nanomaterials, Upton, NY 11973 USA

[b] Current Address: US Air Force Res Lab, Energetic Materials Branch, Eglin AFB, FL 32542 USA

[c] Current Address: Department of Materials Science and Engineering, Chungman National University, 99 Daehak-ro,
Yuseong-gu, Daejeon 34134 Republic of Korea

*Corresponding author. Tel.: +1-805-893-3393; fax: +1-805-893-4120.

E-mail address: buratto@chem.ucsb.edu

Supporting Information

STM investigations on the adsorption and stabilization of water on rutile TiO_2 surfaces, decorated with size-selected V, V_2 and VO_2 clusters were carried out and shown in the supplemental figure below. The soft-landing deposition and STM experiments were carried out under the same conditions detailed in the experimental section of the manuscript. The micrographs here-in were processed with WSxM 5.0 (Nanotech) software.[1]

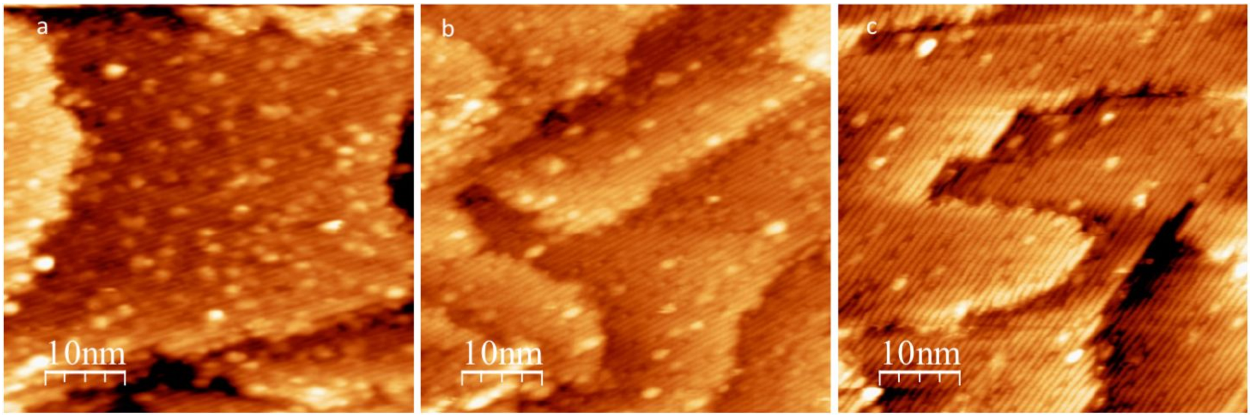

Figure S1. Selected micrographs (50 × 50 nm²) of the rutile TiO_2 (110)-(1× 1) surface decorated with size-selected moieties. (a) V atoms 20 hours after deposition. (b) V_2 clusters 8 hours after deposition. (c) VO_2 clusters 30 hours after deposition. All three systems showed no measurable water adsorption after long exposures to water present in the background.

[1] I. Horcas, R. Fernandez, J. M. Gomez-Rodriguez, J. Colchero, J. Gomez-Herrero, and A. M. Baro, Review of Scientific Instruments 78, 013705 (2007).

Ball and stick models of the lowest energy DFT structures of VO, VO + $1H_2O$, VO + $2H_2O$ and VO + $3H_2O$ bound to a rutile TiO_2 surface.

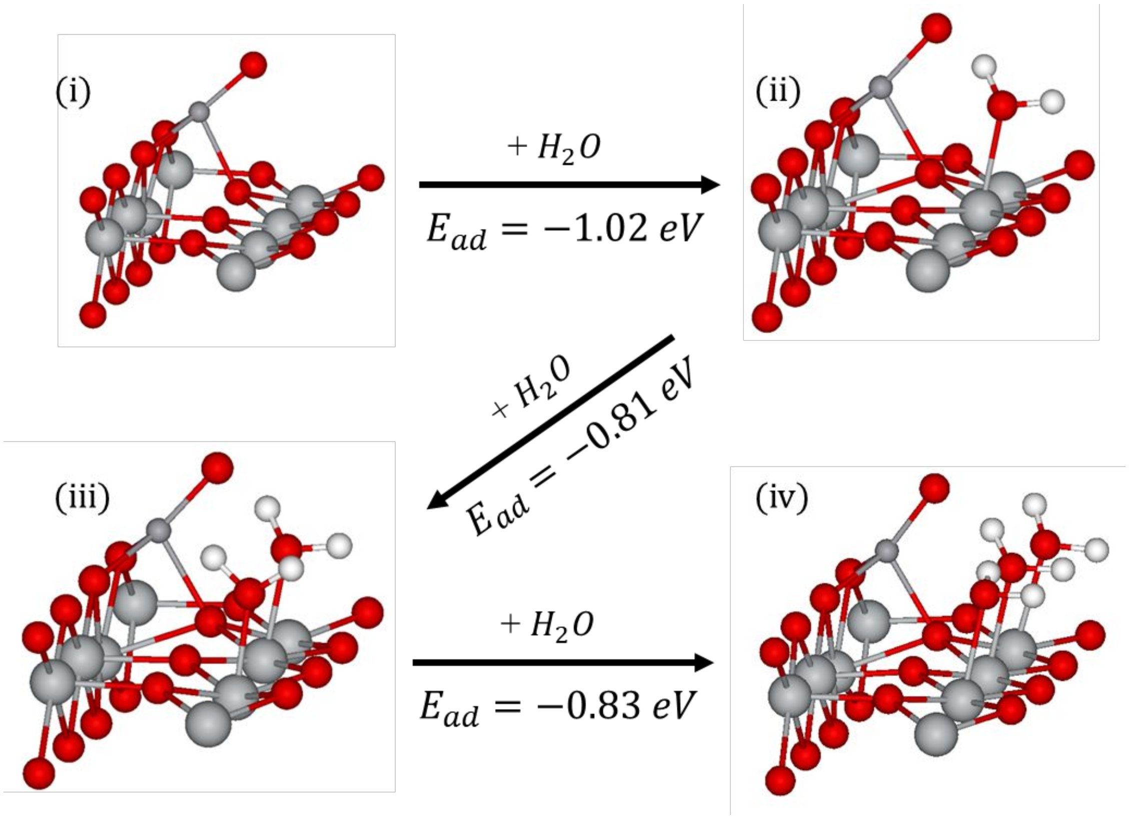

Figure S2 Ball and stick models of the lowest energy structures of water bound to the VO-decorated rutile TiO_2 (110)-(1× 1) surface. (i) The structure of a single VO cluster. (ii) VO + H_2O with an adsorption energy of 1.02 eV. (iii) VO + H_2O + H_2O with additional adsorption energy of 0.81 eV. (iv) VO + H_2O + H_2O + H_2O with an additional adsorption energy of 0.83 eV. Note that our lowest energy structure has the water molecule intact, not dissociated.
